# Impact of endocrine dysregulation on disability and non-motor symptoms in pediatric onset multiple sclerosis

**DOI:** 10.3389/fneur.2023.1304610

**Published:** 2023-12-07

**Authors:** Justin Abe, Saba Jafarpour, My H. Vu, Devon O'Brien, Natalie K. Boyd, Benjamin N. Vogel, Lina Nguyen, Kelli C. Paulsen, Laura E. Saucier, Nusrat Ahsan, Wendy G. Mitchell, Jonathan D. Santoro

**Affiliations:** ^1^John A. Burns School of Medicine, University of Hawaii, Honolulu, HI, United States; ^2^Division of Neurology, Department of Pediatrics, Children's Hospital Los Angeles, Los Angeles, CA, United States; ^3^Biostatistics and Data Management Core, Children's Hospital Los Angeles, Los Angeles, CA, United States; ^4^Department of Neurology, Keck School of Medicine of USC, Los Angeles, CA, United States

**Keywords:** pediatric, multiple sclerosis, hormones, endocrine, depression, fatigue

## Abstract

**Background:**

Pediatric onset multiple sclerosis (POMS) commonly occurs at the time of various endocrine changes. Evaluation of the impact of endocrine status on disease severity in POMS has not been previously explored.

**Objective:**

This study sought to evaluate if sex and stress hormones in children with POMS impact motor and non-motor diseases severity.

**Methods:**

A single-center case control study was performed. Individuals with POMS were compared to individuals without neurologic disease. Each individual had three blood draws assessing stress and sex hormones between 07:00 and 09:00. Measures of fatigue (Epworth sleepiness scale), depression (PHQ-9), and quality of life (PedsQL) assessed at each visit.

**Results:**

Forty individuals with POMS and 40 controls were enrolled. Individuals with POMS had lower free testosterone (*p* = 0.003), cortisol (*p* < 0.001), and ACTH (*p* < 0.001) and had higher progesterone (*p* = 0.025) levels than controls. Relapses and EDSS were not impacted by endocrine variables. The POMS cohort had a significantly higher Epworth score (*p* < 0.001), PHQ-9 score (*p* < 0.001), and lower PQL score (*p* < 0.001) than controls. Non-motor measures were not associated with endocrine status.

**Conclusion:**

Free testosterone, cortisol, ACTH, and progesterone were abnormal in children with POMS although there was no association between endocrine status and markers of disease severity or non-motor symptoms of MS.

## Introduction

Multiple sclerosis (MS) is a chronic inflammatory disease of the central nervous system (CNS) characterized by inflammation, demyelination, and gliosis (1). Roughly 3–5% of MS cases occur in individuals younger than 18 years old, with the majority of these cases occurring in children aged 13 to 16 (1). Although pediatric onset multiple sclerosis (POMS) is fundamentally similar to adult MS, there are some distinct differences which differentiate the two conditions. For instance, whereas 84% of adults with MS present with a relapsing-remitting course, while 98% of POMS patients present with a relapsing-remitting course (2). Younger children with MS typically present with multifocal symptoms, but it remains more common for adolescent patients to present with a single focal symptom, like in adult MS (3). Additionally, although it takes longer for POMS patients to accrue disability, they reach irreversible disability states at a younger age than individuals with adult onset MS (4–6).

**Figure 1:**
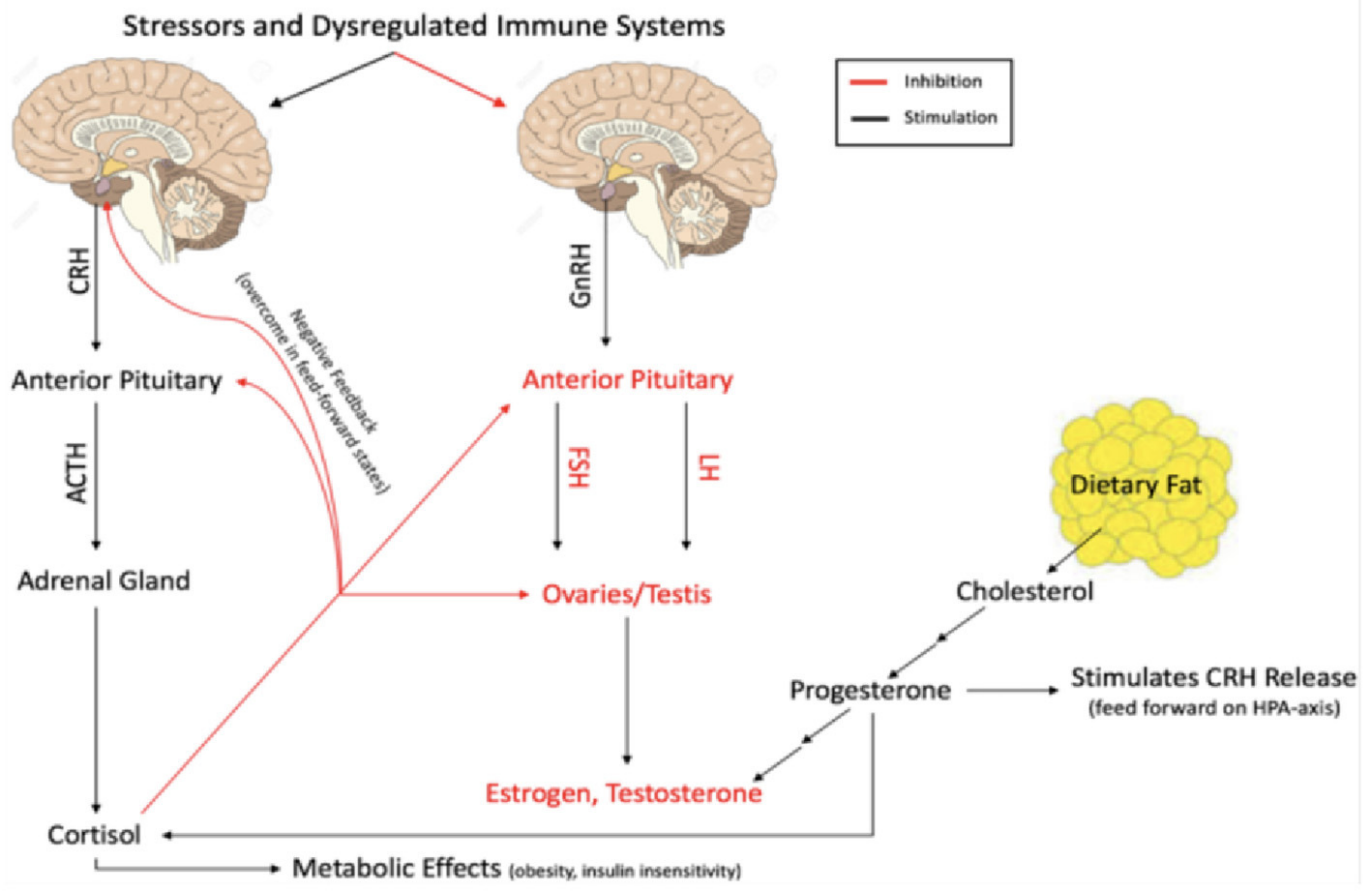
Flow diagram of interaction between the HPA-axis sex hormone production.

Hypothalamic-pituitary-adrenal (HPA) axis dysfunction remains common in adults with MS (7, 8). Studies have linked endocrine dysregulation (typically assessed through cortisol status) to male infertility (9–11), female infertility (12–14), symptomatic hyperprolactinemia (15–17), fatigue (18–20), hyperarousal states (21), depression (19, 22), cognitive impairment (23–25), and most importantly, poorer clinical outcomes in adults with MS (6, 26–29). The HPA-axis is linked to the production of sex hormones through negative feedback mechanisms (Figure 1). Studies in adults have also demonstrated that estrogen, testosterone and progesterone may be protective against relapses and promote remyelination, with the potential to be manipulated to augment disease when combined with disease modifying therapy (DMT) (30–35). Although little is known about the role of sex-hormones in POMS, previous publications have revealed a lack of gender differences in children with POMS until after puberty, suggesting that sex hormones are related to the pathogenesis of POMS (36, 37). Additionally, multiple natural history studies of POMS have identified that obesity, a condition with significant endocrine aspects, and early onset puberty predict earlier onset of MS (38–40).

The role of endocrine dysfunction has been heterogeneously investigated in adults with MS but has not been thoroughly investigated its role children with POMS. The first objective of this study aims to explore the baseline endocrine state of children with POMS using HPA-axis and sex hormones, to determine if these profiles are associated with markers of disease severity and disability. The second objective of this study explores if endocrinologic biomarkers are associated with non-motor symptoms of MS such as fatigue, depression, and sleep dysregulation. We hypothesized that diminished sex hormone and elevated HPA-axis hormone status would be predictive of more severe disease and increased relapses, both clinically and radiographically, and that abnormal sex and HPA-axis hormone states will increase the likelihood and severity of fatigue, depression, and sleep dysregulation.

## Materials and methods

### Approvals and data availability

This study was approved by the Children's Hospital Los Angeles institutional review board (IRB). All participants were required to provide assent when aged <18 years and consent when aged over 18 years. Participants <18 years required a consenting family member or guardian for enrollment. Participants who turned 18 years old during the study were consented at the next follow up visit.

Data from this study is available to qualified investigators pending study team and IRB approval.

### Study population

Participants with POMS were identified through ICD-10 codes (G35) in the electronic medical record system at the host institution. Participants and families/caregivers were approached about participation in research by the study team after regularly scheduled clinical visits. The control cohort was identified through an institutional primary care clinic where patients who were receiving blood-work as part of standard of care evaluation were asked to participate in the study. The control cohort was identified after the POMS cohort had participated in order to optimize age, sex, and Tanner-stage matching.

### Inclusion and exclusion criteria

For individuals with POMS, inclusion criteria involved being between 8 and 18 years of age at the time of diagnosis of POMS, in addition to both meeting McDonald's 2017 criteria (41) and International Pediatric Multiple Sclerosis Study Group (IPMSSG) criteria (42) for POMS. Exclusion criteria included active myelin oligodendrocyte glycoprotein (MOG) antibody positivity of any titer level at the time of enrollment, aquaporin-4 antibody positivity, prior receipt of chemotherapy (including cyclophosphamide), use of finasteride, use of over the counter or prescription hormonal supplements, or history of GU disease (e.g., undescended testis). Non-hormonal gender transition was not exclusionary for this study. Participants who subsequently became pregnant in this study were excluded from further analysis although earlier data points were used.

A control cohort of individuals without neurologic disease was used as a comparator group for this study. Inclusion criteria included an age between 8 and 18 years and receiving primary health care within our institution. Individuals were excluded if they had any chronic medical conditions, prior or active neurological conditions, or had a history of endocrine dysfunction of any kind identified on review of ICD-10 coding for well child visits and chart review. Individuals who were receiving clinical endocrine screening (for sex and stress hormone labs in this study) were also excluded. If consenting to participate in the study, research endocrine labs were added on to existing blood work when possible (e.g., labs would be added to a scheduled CBC for an anemia evaluation) although this was not possible in all cases.

### Laboratory obtainment

During scheduled clinical blood draws, researchers assessed participants had their sex hormones, including luteinizing hormone (LH), follicle stimulating hormone (FSH), prolactin, estrogen, free and total testosterone, and their stress hormones, including cortisol, adrenocorticotropic hormone (ACTH), growth hormone (GH) and progesterone, assessed. When possible, research blood draws were combined with clinical blood draws to minimize venipuncture. Study participants received blood draws at enrollment, three months after the initial encounter, and 6 months after the initial encounter. Labs were obtained in the morning (07:00–09:00 local time) only. Female participants were required to record their last menstrual period prior to blood draw. To avoid variations in the HPA-axis hormonal analysis, labs were not drawn within 8 weeks after the use of any oral or intravenous steroid agent. The timing of all labs received by control participants was identical to that of the POMS cohort.

### Clinical data and disease severity assessments

Demographics including age, gender, race/ethnicity were collected for all participants. For individuals with POMS, disease severity was assessed by the expanded disability severity scale (EDSS), 25-foot walk test, symbol digit modality test (SDMT) and 9-hole peg test. Fatigue, sleep dysregulation, and depression were also assessed using the PediatricQL Multidimensional Fatigue Scale (PedsQL), Epworth Sleepiness Scale (ESS), and the Patient Health Questionnaire-9 (PHQ-9) respectively. To optimize consistency, all neurobehavioral measures were administered by the same research-trained clinical research coordinator at each visit.

All participants received routine, standard of care, neuroimaging following the diagnosis of POMS. The most recent neuroimaging (up to 12 months prior to enrollment of the study) was used as the “baseline” scan. Lesion burden was assessed by manual quantification of white matter lesions. Subsequent scans were obtained during the routine course of practice between 6 and 12 months. Participants with any concern for relapse were imaged per clinical guidelines. Determination of radiographic disease activity in relapse was made by a pediatric-trained neuroradiologist and the principal investigator.

### Statistical analysis

Demographics and characteristics were summarized for the full cohort and across control and POMS groups using mean and standard deviation (SD). Categorical variables were described as a frequency and percentage. Wilcoxon rank-sum tests were used to test for differences in continuous variables and Fisher's exact tests were used to test for associations in categorical variables. Graphical representations (e.g., box plots and correlation graphs) depicted mean outcome values across three visits. To make most efficient use of all follow-up data (visits 1–3), all study analyses utilized linear mixed models with a random participant effect to account for within participant correlation. *P*-values for all corresponding hypothesis tests in the analyses were controlled with respect to false discovery rate (FDR) using Benjamini–Hochberg's procedure. We defined statistical significance using a two-sided FDR adjusted *p* < 0.05. All statistical analyses were conducted in R Studio 4.2.2.

## Results

### Participants' characteristics

Forty individuals with POMS and 40 control individuals without POMS were included in this study. Participants' demographics and clinical characteristics are reported in Table 1. In the POMS cohort, the mean (SD) age at diagnosis was 14.4 (1.7) years and mean (SD) age at first therapy was 14.9 (2) years. Mean (SD) duration of symptoms was 2.5 (1.7) years.

**Table 1:** Demographics and phenotypes of the study cohort.

**Characteristic**	**All Patients (*n* = 80)^a^**	**Control (*n* = 40)^a^**	**POMS (*n* = 40)^a^**	***P* ^b^**
**Age at testing (years)**	16.9 (2.3)	17.0 (2.2)	16.8 (2.4)	0.8
**Sex**				0.9
Male	30 (38%)	15 (38%)	15 (38%)	
Female	50 (62%)	25 (62%)	25 (62%)	
**Race**				0.5
White	66 (82%)	31 (78%)	35 (88%)	
Black	8 (10%)	5 (12%)	3 (8%)	
Asian	6 (8%)	4 (10%)	2 (5%)	
**Ethnicity**				0.4
Hispanic	43 (54%)	19 (48%)	24 (60%)	
Non-Hispanic	37 (46%)	21 (52%)	16 (40%)	
**Tanner Stage**				0.09
3	1 (1%)	0 (0%)	1 (3%)	
4	10 (13%)	8 (20%)	2 (5.0%)	
5	69 (86%)	32 (80%)	37 (92%)	
**Age at diagnosis (years)**	-	-	14.4 (1.7)	-
**Age at 1**^**st**^ **therapy (years)**	-	-	14.9 (2)	-
**Duration of symptoms (years)**	-	-	2.5 (1.7)	-

a Mean (sd), n (%).

b Fisher's exact test; Wilcoxon rank sum test.

### Difference in hormones between groups

**Figure 2:**
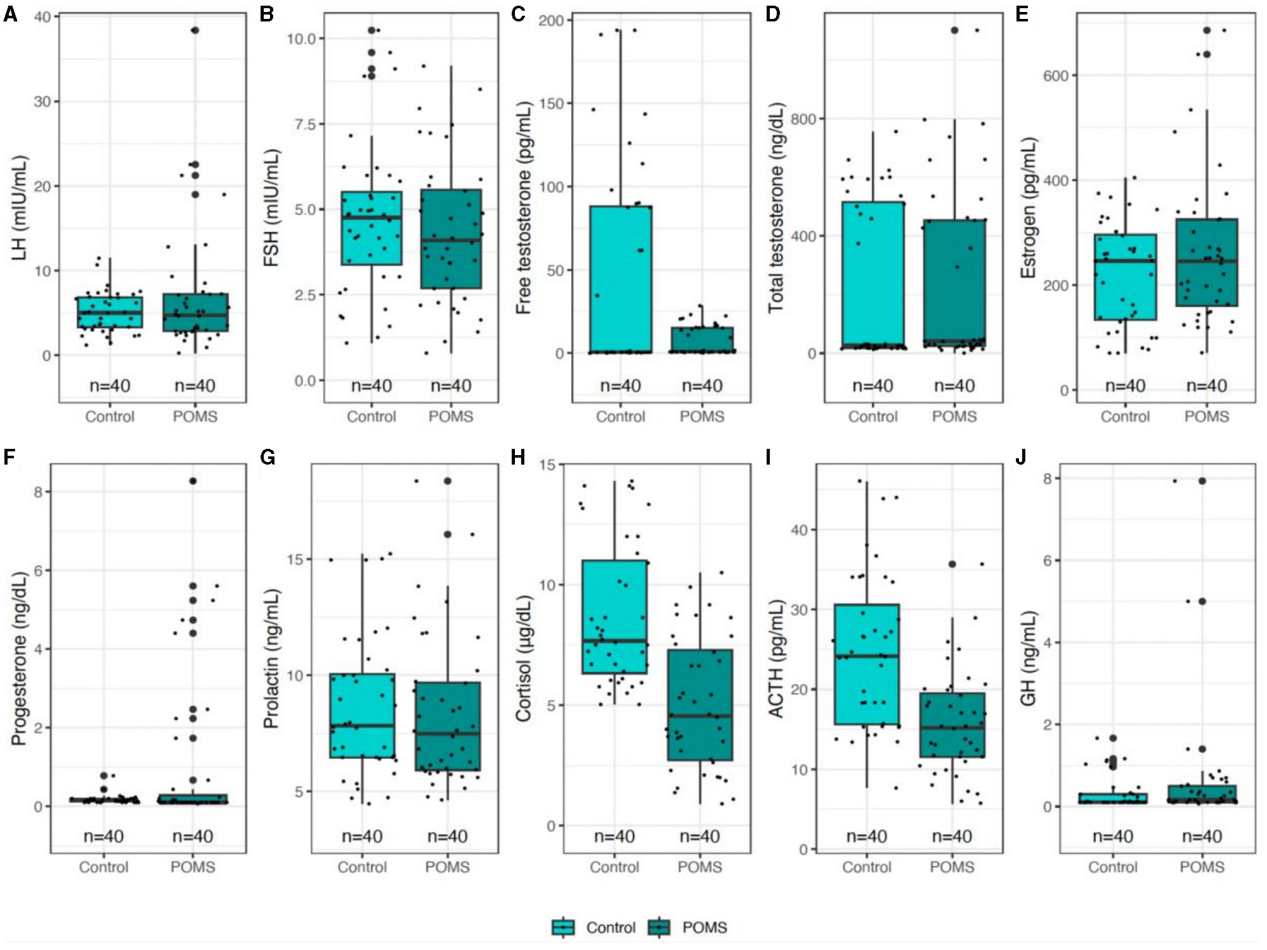
Differences in hormones between POMS and control.

There was no significant difference in LH, FSH, estrogen, total testosterone, prolactin, and GH between groups. Participants with POMS had a significantly lower free testosterone (*p* = 0.003), cortisol (*p* < 0.001), and ACTH (*p* < 0.001) level than the control group and a significantly higher progesterone (*p* = 0.025) level than the control group (Figure 2, Table 2).

**Table 2:** Differences in hormones between POMS and control.

**Hormones**	**Control^a^**	**POMS^a^**	**POMS—control^a^**	**P**
LH (mIU/mL)	5.08 (4.3, 5.8)	6.9 (4.6, 9.2)	1.82 (−0.6, 4.2)	0.232
FSH (mIU/mL)	4.71 (4, 5.4)	4.33 (3.7, 5)	−0.38 (−1.3, 0.6)	0.546
Estrogen (pg/mL)	220.12 (189.2, 251.1)	262.88 (218.5, 307.1)	42.55 (−10.7, 95.7)	0.232
Free testosterone (pg/mL)	43.26 (23.3, 63.2)	7.3 (4.6, 10)	−35.97 (−55.8, −16.1)	**0.003**
Total testosterone (ng/dL)	223.77 (139, 308.6)	232.46 (140.6, 324.6)	8.27 (−115, 131.7)	0.896
Progesterone (ng/dL)	0.18 (0.1, 0.2)	0.99 (0.4, 1.6)	0.81 (0.2, 1.4)	**0.025**
Prolactin (ng/mL)	8.61 (7.7, 9.6)	8.32 (7.3, 9.3)	−0.29 (−1.7, 1.1)	0.751
Cortisol (μg/dL)	8.69 (7.8, 9.6)	5.06 (4.2, 5.9)	−3.63 (−4.9, −2.4)	**< 0.001**
ACTH (pg/mL)	24.7 (21.7, 27.7)	15.71 (13.7, 17.7)	−8.98 (−12.6, −5.4)	**< 0.001**
GH (ng/mL)	0.31 (0.2, 0.4)	0.6 (0.1, 1.1)	0.3 (−0.2, 0.8)	0.306

a Values are presented as mean (95% confidence interval). Bold values indicate *p* < 0.05.

Although some participants with POMS had fluctuating hormone levels over time, the majority of patients had fairly stable hormone levels throughout the three visits (Supplementary Figure 1).

### Demographics and hormones in POMS

**Figure 3:**
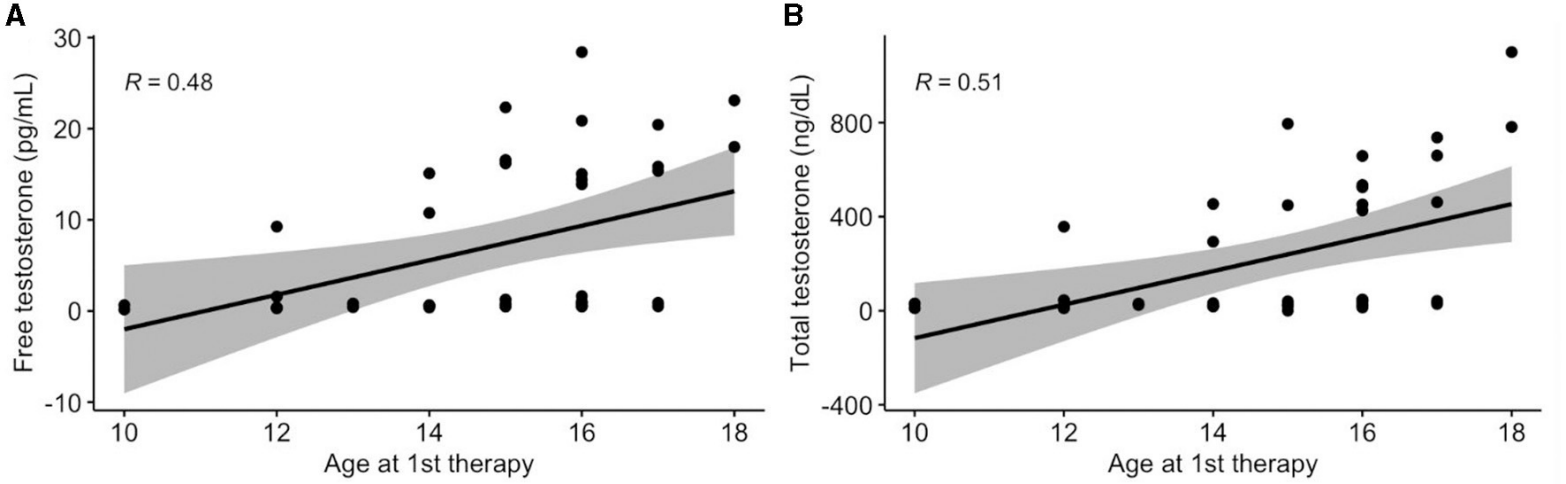
Association between age at first therapy and testosterone. Values presented as Spearman's correlation coefficients.

There were no significant associations between demographics and hormones, with the exceptions of age at first therapy and sex. The association between age at first therapy and testosterone are displayed in Figure 3. Age at first therapy was positively associated with higher free testosterone (β = 1.9; 95% CI: 0.6, 3.2; *p* = 0.007) and higher total testosterone (β = 69.8; 95% CI: 27.4, 112.3; *p* = 0.003).

The difference in hormones based on sex is presented in Table 3. Females had a higher LH (*p* = 0.017), FSH (*p* = 0.004), estrogen (*p* < 0.001), progesterone (*p* = 0.031), and prolactin (*p* = 0.002), and males had a higher free testosterone (*p* < 0.001) and total testosterone (*p* < 0.001).

**Table 3:** Differences in hormones between males and females.

**Hormones**	**Male (95% CI)**	**Female (95% CI)**	**Female–Male (95% CI)**	***P***
LH (mIU/mL)	3.29 (2.3, 4.3)	9.13 (5.7, 12.6)	5.8 (1.5, 10.2)	**0.017**
FSH (mIU/mL)	3.03 (2, 4)	5.12 (4.4, 5.9)	2.1 (0.9, 3.3)	**0.004**
Estrogen (pg/mL)	156.24 (132.3, 180.2)	330.14 (274, 385.6)	173.8 (101.7, 245.6)	**< 0.001**
Free testosterone (pg/mL)	15.69 (12.3, 19.1)	2.28 (0.1, 4.4)	−13.5 (−17.1, −9.8)	**< 0.001**
Total testosterone (ng/dL)	506.61 (399.4, 613.8)	71.14 (−14.5, 156.8)	−444.3 (−562, −325.8)	**< 0.001**
Progesterone (ng/dL)	0.1 (0.09, 0.11)	1.53 (0.6, 2.5)	1.4 (0.3, 2.6)	**0.031**
Prolactin (ng/mL)	6.31 (5.7, 6.9)	9.55 (8.1, 10.9)	3.2 (1.4, 5.1)	**0.002**
Cortisol (μg/dL)	5.78 (4.1, 7.4)	4.62 (3.7, 5.6)	−1.2 (−2.9, 0.6)	0.221
ACTH (pg/mL)	16 (13.2, 18.8)	15.53 (12.7, 18.4)	−0.5 (−4.6, 3.7)	0.825
GH (ng/mL)	0.22 (0.1, 0.3)	0.84 (0.1, 1.6)	0.6 (−0.3, 1.5)	0.221

Bold values indicate *p* < 0.05.

### Hormones and disability in POMS

The median (IQR) baseline vitamin D level in the POMS cohort was 33.50 ng/mL (26.8, 40). There was no association between either sex or stress hormones and vitamin D level. Annualized relapse rate (ARR) and EDSS functional scores were also not significant outcomes across a variety of hormonal estimates. There were no significant associations between EDSS functional scores and hormones in the POMS cohort, with the exceptions of functional cerebellar scores (Supplementary Table 1). Having a functional cerebellar score of at least one was associated with higher free testosterone (mean difference: 10.19; 95% CI: 3.2, 17.2; *p* = 0.035) and higher total testosterone (mean difference: 444.04; 95% CI: 225.8, 663; *p* < 0.001).

Throughout the three visits, 15% and 8% of participants with POMS had lymphopenia and leukopenia, respectively, of participants with POMS. Due to the low prevalence, leukopenia and lymphopenia were not significantly associated with hormonal variation, except for total testosterone. Having leukopenia was associated with a lower total testosterone (mean difference, −171.17; 95% CI, −286.6 to −54.7) (Supplementary Table 2).

### Hormones and non-motor disability in POMS

**Figure 4:**
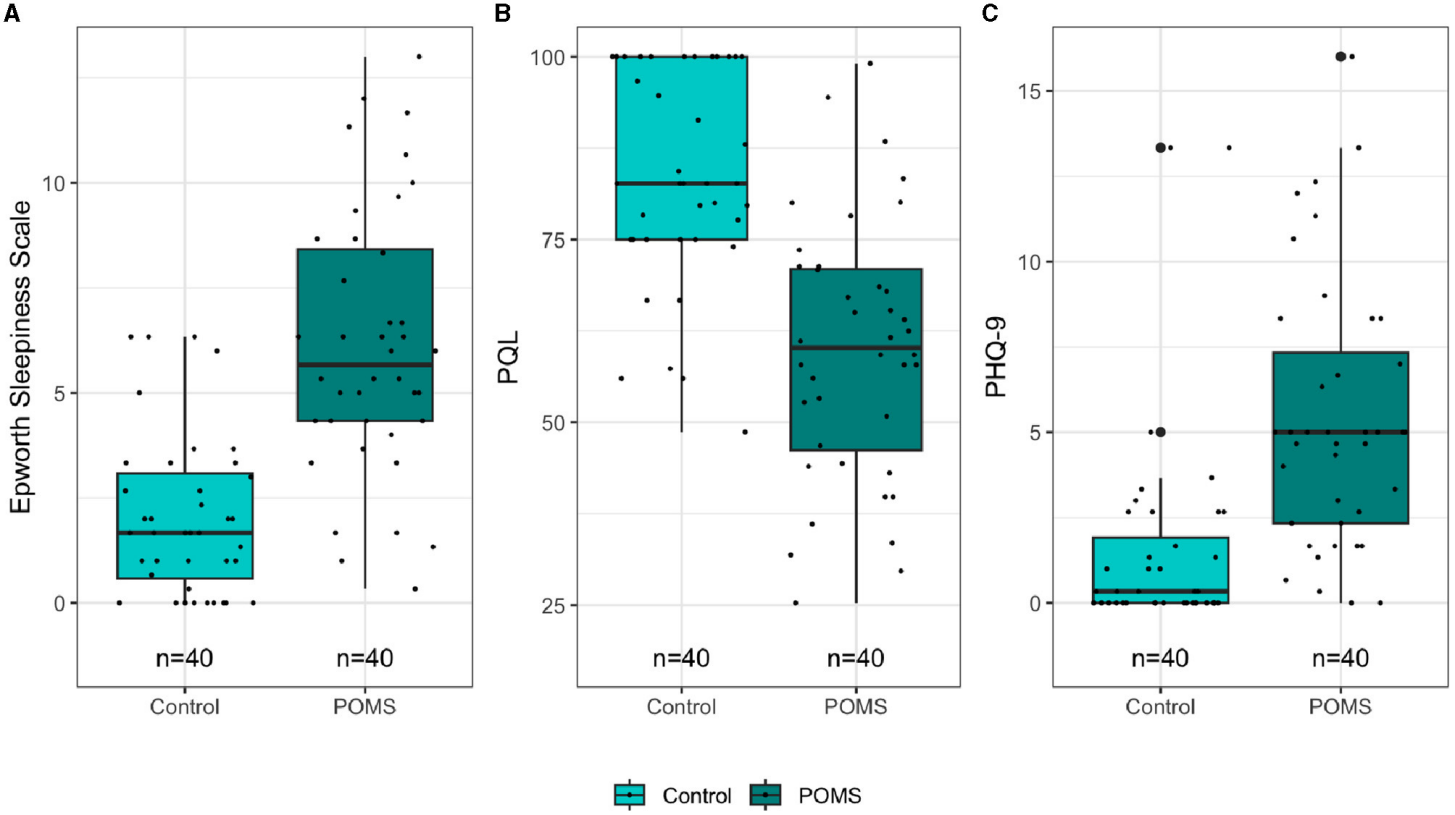
Differences in epworth, PQL, and PHQ9 score between POMS and control.

The differences in ESS, PedsQL, and PHQ-9 scores between POMS and Control cohorts are presented in Figure 4. The POMS cohort had a significantly higher ESS (mean difference = 4.1; 95% CI: 3, 5.3; *p* < 0.001) and PHQ-9 score (mean difference = 3.8; 95% CI: 2.3, 5.4; *p* < 0.001), and a significantly lower PedsQL score (mean difference = −24.3; 95% CI:−31.4,−17.2; *p* < 0.001) than the control cohort. Although some of the controls and POMS patients had fluctuating ESS, PedsQL, and PHQ-9 scores over time, most participants had stable scores across their three visits (Supplementary Figure 2).

The associations between endocrine variables, and ESS, PedsQL, and PHQ-9 are presented in Table 4. There were no significant associations observed between hormones and ESS or PedsQL scores. PHQ-9 score was significantly associated with a higher progesterone (*p* < 0.001, 95%CI: 0.14–0.61) in the POMS cohort, but this association was not significantly different from that of the control cohort.

**Table 4:** Associations between endocrine variables and non-motor measures fatigue/sleepiness, depression and quality of life.

**Hormones**	**Control (95% CI)**	**P**	**POMS (95% CI)**	**P**	**POMS–control (95% CI)**	**P**
**Fatigue/sleepiness (Epworth Sleepiness Score)**						
LH (mIU/mL)	−0.06 (−0.2, 0.1)	0.90	0.03 (−0.01, 0.07)	0.33	0.09 (−0.07, 0.25)	0.671
FSH (mIU/mL)	−0.13 (−0.3, 0.07)	0.90	0.23 (0.03, 0.42)	0.20	0.36 (0.07, 0.65)	0.100
Estrogen (pg/mL)	0 (0, 0.01)	0.94	0 (0, 0)	0.34	0 (−0.01, 0)	0.671
Free testosterone (pg/mL)	0 (−0.01, 0.01)	0.94	−0.01 (−0.1, 0.08)	0.90	−0.01 (−0.1, 0.08)	0.850
Total testosterone (ng/dL)	0 (0, 0)	0.90	0 (0, 0)	0.84	0 (0, 0)	0.671
Progesterone (ng/dL)	−0.29 (−2.5, 1.93)	0.94	0.06 (−0.15, 0.27)	0.84	0.34 (−1.89, 2.57)	0.850
Prolactin (ng/mL)	−0.09 (−0.27, 0.09)	0.90	−0.01 (−0.15, 0.14)	0.92	0.08 (−0.14, 0.31)	0.671
Cortisol (μg/dL)	0 (−0.12, 0.11)	0.95	0.11 (−0.02, 0.24)	0.33	0.11 (−0.07, 0.29)	0.671
ACTH (pg/mL)	−0.02 (−0.07, 0.02)	0.90	−0.07 (−0.13, 0)	0.30	−0.04 (−0.12, 0.04)	0.671
GH (ng/mL)	−0.28 (−1.73, 1.16)	0.94	−0.14 (−0.43, 0.15)	0.58	0.14 (−1.33, 1.62)	0.850
**Pediatric Quality of Life (PedsQL)**						
LH (mIU/mL)	0.21 (−0.45, 0.88)	0.83	−0.05 (−0.22, 0.13)	0.74	−0.26 (−0.95, 0.43)	0.750
FSH (mIU/mL)	0.21 (−0.73, 1.15)	0.83	−0.17 (−1.03, 0.69)	0.78	−0.38 (−1.66, 0.89)	0.787
Estrogen (pg/mL)	0 (−0.02, 0.03)	0.88	0 (−0.01, 0.02)	0.68	0 (−0.02, 0.03)	0.989
Free testosterone (pg/mL)	−0.04 (−0.1, 0.03)	0.83	−0.45 (−0.94, 0.02)	0.20	−0.42 (−0.91, 0.07)	0.567
Total testosterone (ng/dL)	0 (−0.01, 0.01)	0.83	−0.01 (−0.02, 0)	0.40	−0.01 (−0.3, 0) 0.01	0.750
Progesterone (ng/dL)	−4.8 (−13.78, 4.14)	0.83	−0.9 (−1.79, 0)	0.20	3.91 (−5.08, 12.93)	0.750
Prolactin (ng/mL)	0.19 (−0.63, 1.01)	0.83	0.18 (−0.45, 0.81)	0.74	−0.01 (−1.05, 1.03)	0.990
Cortisol (μg/dL)	0.25 (−0.25, 0.74)	0.83	−0.32 (−0.86, 0.23)	0.50	−0.56 (−1.3, 0.17)	0.567
ACTH (pg/mL)	0.05 (−0.13, 0.24)	0.83	0.3 (0, 0.6)	0.20	0.25 (−0.11, 0.6)	0.567
GH (ng/mL)	−1.62 (−8.52, 5.26)	0.83	0.07 (−1.2, 1.35)	0.91	1.69 (−5.31, 8.71)	0.787
**Depression (PHQ-9)**						
LH (mIU/mL)	−0.05 (−0.23, 0.13)	0.57	0.01 (−0.04, 0.06)	0.91	0.06 (−0.12, 0.25)	0.712
FSH (mIU/mL)	−0.01 (−0.26, 0.23)	0.92	0.03 (−0.2, 0.26)	0.92	0.04 (−0.3, 0.38)	0.900
Estrogen (pg/mL)	0 (0, 0.01)	0.92	0 (0, 0)	0.95	0 (−0.01, 0)	0.712
Free testosterone (pg/mL)	0 (−0.02, 0.01)	0.92	−0.07 (−0.18, 0.05)	0.50	−0.06 (−0.17, 0.05)	0.712
Total testosterone (ng/dL)	0 (0, 0)	0.92	0 (0, 0)	0.91	0 (0, 0.01)	0.712
Progesterone (ng/dL)	−0.45 (−2.88, 1.97)	0.92	0.38 (0.14, 0.61)	< 0.001	0.83 (−1.6, 3.27)	0.712
Prolactin (ng/mL)	0.03 (−0.18, 0.23)	0.92	0.1 (−0.06, 0.27)	0.50	0.08 (−0.19, 0.34)	0.712
Cortisol (μg/dL)	−0.06 (−0.19, 0.07)	0.92	0.1 (−0.05, 0.24)	0.50	0.16 (−0.04, 0.36)	0.550
ACTH (pg/mL)	−0.01 (−0.05, 0.04)	0.92	−0.09 (−0.16, −0.01)	0.15	−0.08 (−0.17, 0.01)	0.550
GH (ng/mL)	−0.17 (−1.9, 1.57)	0.92	−0.05 (−0.39, 0.28)	0.92	0.11 (−1.66, 1.88)	0.900

## Discussion

This study demonstrates that children with POMS present lower levels of free testosterone, cortisol, and ACTH, in addition to higher levels of progesterone, when compared to age, weight, sex, and Tanner Stage matched controls. Previous studies have identified relationships between significant endocrine events and POMS pathogenesis (36–40), but, to our knowledge, this is the first study to identify these endocrinologic differences in children with POMS.

Previous studies suggest that a relationship between sex hormones and early onset of disease in POMS exists, though date from our cohort demonstrated no associations between endocrine dysregulation and earlier age of onset of this condition (36, 37). This may be associated with high Tanner stages within the POMS group wherein 92% of the POMS cohort were Tanner Stage 5 at the time of study participation. It is possible that this effect may have been observed if hormones were taken closer to the time of original diagnosis as the mean duration of symptoms in our study was 2.5 years. Although age at diagnosis was not predictive of any hormonal associations, age at first therapy was positively associated with a higher level of free and total testosterone. This may indicate that higher levels of testosterone may be protective in preventing and earlier utilization of disease modifying therapy in this population although this will require further longitudinal study to properly evaluate.

Interestingly, BMI was not predictive of any endocrine abnormalities in our study, which conflicts with previous studies that found that higher BMI was predictive of an earlier onset of POMS (38, 40). This may be because BMI is a relatively limited metric and not an accurate assessment of adiposity. One explanation for the connection between BMI and POMS is that adipokines may play a role in pathogenesis (43). Thus, if patients with a high BMI in our study had an inconsistent level of adiposity, our results could have been altered or masked a potential effect. Further, vitamin D was not a significant outcome across a variety of endocrine variables. Previous studies suggest that vitamin D plays a role in the onset, progression, and relapse of MS (44), as well as in the onset and relapse rate of POMS (45, 46). It is possible that the mechanism in which vitamin D is involved in POMS pathogenesis is distinct from the role of hormones in POMS pathogenesis although the limited power of this study may have also been unable to detect differentiation.

Our results also suggested that relapses (defined by ARR) and disability (defined by EDSS) were not impacted by endocrine status. When EDSS functional scores were evaluated, cerebellar scores had a significant positive association with testosterone levels. However, as the number of individuals with an abnormal functional cerebellar score was very low, the validity of these associations is uncertain. This also applies to the relationship observed between leukopenia and total testosterone, as the prevalence of leukopenia in our study was very low. Individuals with POMS displayed skewed disability scores (lower EDSS) and lower relapses (lower ARR) and the lack of distribution of data may have contributed to our non-significant findings. This is consistent with another study which suggests that POMS patients tend to have lower EDSS scores than adult-onset MS patients and that the stepwise nature of EDSS scoring may limit the description of the POMS disease status (4).

Individuals with POMS had a significantly higher ESS and PHQ-9 score, and a significantly lower PedsQL score, indicating a higher level of sleepiness and depression and lower quality of life. There were no clinically significant associations between endocrine status and any of the neurocognitive measures. This was surprising given the high rates of fatigue and depression and lower quality of life in the POMS cohort and may be reflective or either low study power or inferior testing instruments given they are all screening, rather than diagnostic, measures. Previous studies in adult MS populations have suggested that HPA-axis hyperactivity is associated with increased fatigue and depression (18–20, 22), but in our study, we observed that individuals with POMS had a significantly lower free cortisol and ACTH level, suggesting HPA-axis underactivity. The mechanism in which individuals with POMS develop fatigue and depression may be different than that of adults with the condition and represents an important, and potentially modifiable, area of investigation for future studies.

This study is not without limitations. Although the study involved an age, sex, and Tanner-stage matched control cohort of individuals without POMS, the sample size for both groups was small, limiting the power to detect differences between groups. Regardless, generalizability in this cohort to other patient populations is difficult although our sample size was comparable to other previous POMS studies (36, 39, 40, 43). Our study was a single-center study which introduces ascertainment and severity bias as our site is the only regional center for POMS care in the region. In addition, the generalizability of these data may also be limited by the single center design. As the median time between the diagnosis of POMS and this study was 2.5 years, nearly all patients were already Tanner 4 or 5 at the time of participation. As such, endocrine assessments are more reflective of mature, post-pubertal, samples. Evaluating these endocrine labs would be very beneficial at the time of diagnosis and is being planned as a subsequent study. Sex and stress hormones are also notoriously variable and while sample collection occurred in a standardized fashion at the same time for each blood draw, there was variability in the read outs. For this reason, an average level (across three collections) was used for calculations in this study to minimize outlier data. Screening measures such as the PHQ-9, PedsQL, and ESS were utilized in this study as opposed to more comprehensive assessments of depression, quality of life and fatigue and these briefer testing batteries may not have captured clinically significant effects. Future studies with more in-depth cognitive testing are already underway on this study group to further explore this potential association.

## Conclusions and future directions

This study identified significant differences in baseline free testosterone, cortisol, ACTH, and progesterone in children with POMS although there was not a clear connection between hormones and markers of disease severity or non-focal symptoms of MS. Future research should investigate the clinical implication of these hormonal differences in POMS and the potential association with neurocognitive, non-motor, clinical symptoms.

## Data availability statement

The raw data supporting the conclusions of this article will be made available by the authors, without undue reservation.

## Ethics statement

The studies involving humans were approved by Children's Hospital Los Angeles and Keck School of Medicine of USC. The studies were conducted in accordance with the local legislation and institutional requirements. Written informed consent for participation in this study was provided by the participants' legal guardians/next of kin.

## Author contributions

JA: Data curation, Methodology, Validation, Writing – original draft, Writing – review & editing. SJ: Data curation, Formal analysis, Investigation, Methodology, Writing – original draft, Writing – review & editing. MV: Data curation, Formal analysis, Methodology, Validation, Writing – original draft, Writing – review & editing. DO'B: Data curation, Formal analysis, Investigation, Writing – original draft, Writing – review & editing. NB: Data curation, Validation, Writing – original draft, Writing – review & editing. BV: Data curation, Validation, Visualization, Writing – original draft, Writing – review & editing. LN: Data curation, Investigation, Validation, Writing – original draft, Writing – review & editing. KP: Data curation, Investigation, Methodology, Supervision, Validation, Writing – original draft, Writing – review & editing. LS: Conceptualization, Supervision, Validation, Writing – original draft, Writing – review & editing. NA: Project administration, Supervision, Validation, Writing – review & editing. WM: Investigation, Methodology, Project administration, Supervision, Validation, Writing – review & editing. JS: Conceptualization, Formal analysis, Funding acquisition, Investigation, Methodology, Project administration, Resources, Supervision, Validation, Writing – original draft, Writing – review & editing.
