# Supplementary material for: Impact of endocrine dysregulation on disability and non-motor symptoms in pediatric onset multiple sclerosis
Source: Front Neurol. 2023 Dec 7;14:1304610. doi: 10.3389/fneur.2023.1304610 (PMC10733457; doi:10.3389/fneur.2023.1304610)
Supplement: Supplementary file 1 [file Table_1.DOCX]

**Supplementary Table 1:** Differences in Hormones Between ARR Score Groups

|  | **ARR** | | **EDSS** | | **FS CBL** |  |
| --- | --- | --- | --- | --- | --- | --- |
| **Hormones** | **At least 1 - Less than 1**^a^ | **p-value** | **At least 1 - Less than 1**^a^ | **p-value** | **At least 1 - less than 1**^a^ | **p-value** |
| LH (mIU/mL) | 2.2 (-2.2, 6.65) | 0.621 | 3.83 (-1.1, 8.73) | 0.338 | -4.51 (-11.1, 2.1) | 0.310 |
| FSH (mIU/mL) | 0.73 (-0.4, 1.8) | 0.621 | 0.28 (-1.2, 1.72) | 0.835 | -1.95 (-3.7, -0.1) | 0.120 |
| Estrogen (pg/mL) | -38.46 (-116.4, 39.56) | 0.621 | -77.6 (-170.8, 15.92) | 0.338 | -126.64 (-248.5, -4.9) | 0.120 |
| Free testosterone (pg/mL) | -0.07 (-1.6, 1.46) | 0.974 | 0.57 (-3.7, 4.86) | 0.835 | 10.19 (3.2, 17.2) | **0.035** |
| Total testosterone (ng/dL) | -1.73 (-69.7, 67.57) | 0.974 | 17.64 (-147.1, 182.22) | 0.835 | 444.04 (225.8, 663) | **< 0.001** |
| Progesterone (ng/dL) | -0.59 (-1.6, 0.42) | 0.621 | -1.02 (-2.3, 0.27) | 0.338 | -1.03 (-2.8, 0.7) | 0.357 |
| Prolactin (ng/mL) | -0.03 (-1.5, 1.49) | 0.974 | 0.46 (-1.7, 2.63) | 0.835 | -1.52 (-4.4, 1.4) | 0.381 |
| Cortisol (μg/dL) | -0.63 (-2.2, 0.93) | 0.621 | -1.48 (-3.3, 0.35) | 0.338 | -1.83 (-4.3, 0.6) | 0.298 |
| ACTH (pg/mL) | -1.25 (-4.4, 1.88) | 0.621 | -2.29 (-6.6, 2.02) | 0.604 | -2.36 (-8.1, 3.4) | 0.477 |
| GH (ng/mL) | 0.34 (-0.4, 1.07) | 0.621 | 0.39 (-0.6, 1.37) | 0.718 | -0.29 (-1.6, 1.02) | 0.669 |

^a^ Values are presented as mean (95% confidence interval)
