# Supplementary material for: Impact of endocrine dysregulation on disability and non-motor symptoms in pediatric onset multiple sclerosis
Source: Front Neurol. 2023 Dec 7;14:1304610. doi: 10.3389/fneur.2023.1304610 (PMC10733457; doi:10.3389/fneur.2023.1304610)
Supplement: Supplementary file 2 [file Table_2.DOCX]

**Supplementary Table 2:** Differences in Hormones Between EDSS Score Groups

| **Hormones** | **No Leukopenia** | **Leukopenia** | **Leukopenia - No leukopenia (95% CI)** | **p-value** |
| --- | --- | --- | --- | --- |
| LH (mIU/mL) | 6.93 | 6.43 | -0.5 (-10.6, 9.48) | 0.922 |
| FSH (mIU/mL) | 4.28 | 5.32 | 1.04 (-1.2, 3.23) | 0.782 |
| Estrogen (pg/mL) | 266.53 | 194.63 | -71.9 (-235.2, 92.63) | 0.782 |
| Free testosterone (pg/mL) | 7.22 | 8.77 | 1.55 (-1.1, 4.21) | 0.782 |
| Total testosterone (ng/dL) | 241.24 | 70.07 | -171.17 (-286.6, -54.73) | **0.040** |
| Progesterone (ng/dL) | 1.05 | -0.26 | -1.31 (-3.4, 0.83) | 0.782 |
| Prolactin (ng/mL) | 8.35 | 7.7 | -0.65 (-3.7, 2.39) | 0.922 |
| Cortisol (μg/dL) | 5.01 | 6.05 | 1.04 (-2.3, 4.39) | 0.908 |
| ACTH (pg/mL) | 15.75 | 14.86 | -0.89 (-7.2, 5.42) | 0.922 |
| GH (ng/mL) | 0.61 | 0.5 | -0.11 (-1.6, 1.38) | 0.922 |
