# Supplementary figures and images for: Impact of endocrine dysregulation on disability and non-motor symptoms in pediatric onset multiple sclerosis
Source: Front Neurol. 2023 Dec 7;14:1304610. doi: 10.3389/fneur.2023.1304610 (PMC10733457; doi:10.3389/fneur.2023.1304610)

**Supplemental Figure 1.** Change in Hormone Levels over Time in POMS

**
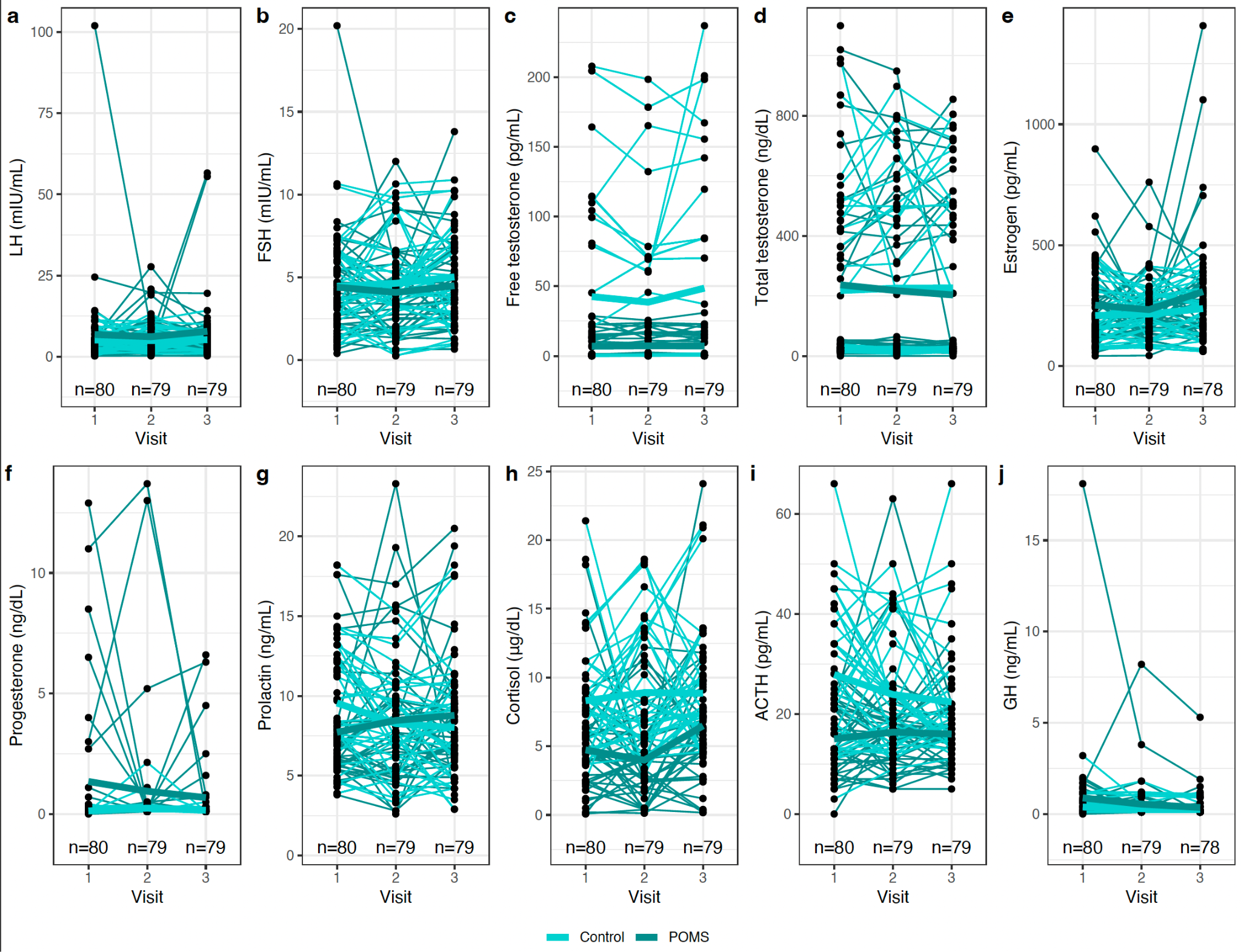
**

Supplement: Supplementary file 3 [file Data_Sheet_1.DOCX]

**Supplemental Figure 2**. Changes in Epworth, PQL, and PHQ9 Score over Time in POMS


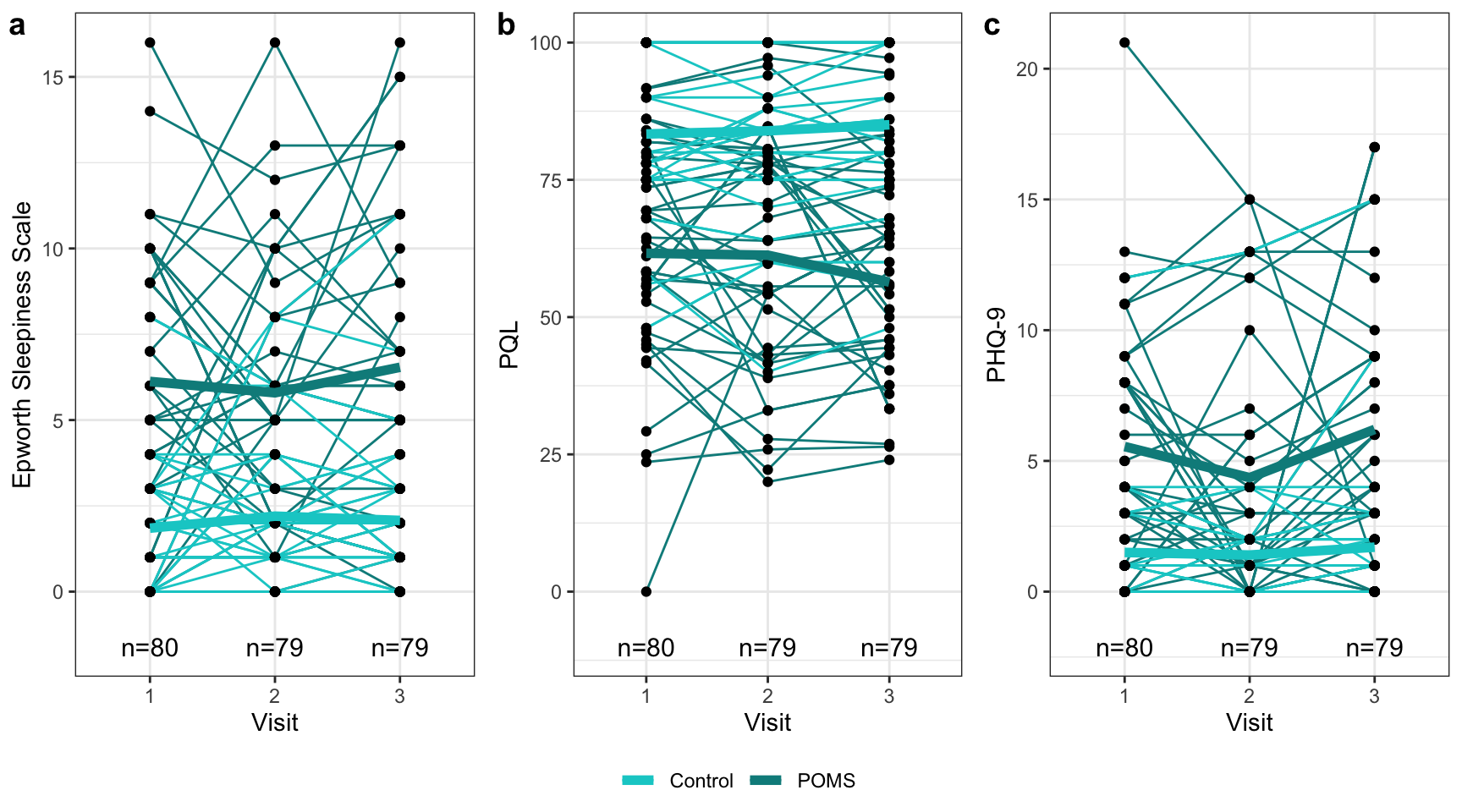

Supplement: Supplementary file 4 [file Data_Sheet_2.DOCX]
